# Chromatin Accessibility and Interactions in the Transcriptional Regulation of T Cells

**DOI:** 10.3389/fimmu.2018.02738

**Published:** 2018-11-22

**Authors:** Peng Li, Warren J. Leonard

**Affiliations:** Laboratory of Molecular Immunology and the Immunology Center, National Heart, Lung, and Blood Institute, National Institutes of Health, Bethesda, MD, United States

**Keywords:** transcription factors, chromatin accessibility, T cells, STAT5, ChIA-PET, chromatin interactions

## Abstract

During T cell differentiation and activation, specific stimuli, and a network of transcription factors (TFs) are involved in orchestrating chromatin accessibility, establishing enhancer-promoter interactions, and regulating gene expression. Over the past few years, there have been new insights into how chromatin interactions coordinate differentiation during T cell development and how regulatory elements are programmed to allow T cells to differentially respond to distinct stimuli. In this review, we discuss recent advances related to the roles of TFs in establishing the regulatory chromatin landscapes that orchestrate T cell development and differentiation. In particular, we focus on the role of TFs (e.g., TCF-1, BCL11B, PU.1, STAT3, STAT5, AP-1, and IRF4) in mediating chromatin accessibility and interactions and in regulating gene expression in T cells, including gene expression that is dependent on IL-2 and IL-21. Furthermore, we discuss the state of knowledge on enhancer-promoter interactions and how autoimmune disease risk variants can be linked to molecular functions of putative target genes.

## Introduction

Transcriptomic profiles determine the phenotype and function of cells, and this process is tightly controlled by various transcription factors (TFs), epigenetics, and chromatin interactions to define transcriptional patterns in response to cellular signals. More specifically, control of gene expression depends not only on the binding of sequence-specific TFs to target DNA sequences, but also on chromatin accessibility, which is controlled by the proper packaging of DNA/nucleosomes (chromatin) within the nucleus, leading to the arrangement of the genome into distinct spatial structures. Differences in chromatin composition can determine gene expression profiles in cells by providing relative accessibility (open or closed) of key regions to TFs that bind to DNA. Within the immune system, upon cellular stimulation by extracellular signals (e.g., via the T cell receptor (TCR) or cytokines), chromatin composition is modified through the concerted actions of signal-specific TFs and chromatin modifiers via a dynamic process. Naturally occurring genetic mutations in binding sites for TFs that alter the chromatin landscape can potentially disrupt or establish chromatin interactions, thereby resulting in altered gene expression profiles, and predisposing to cancer, autoimmune disease, allergy, immunodeficiency, or other immune disorders. In this review, we focus on the cooperative actions of TFs that play critical roles in shaping the chromatin landscape and accessibility in early and mature T cell development, and how these dynamic changes can alter gene expression profiles.

## Transcription factors that establish chromatin landscape during early T cell development

**Figure 1:**
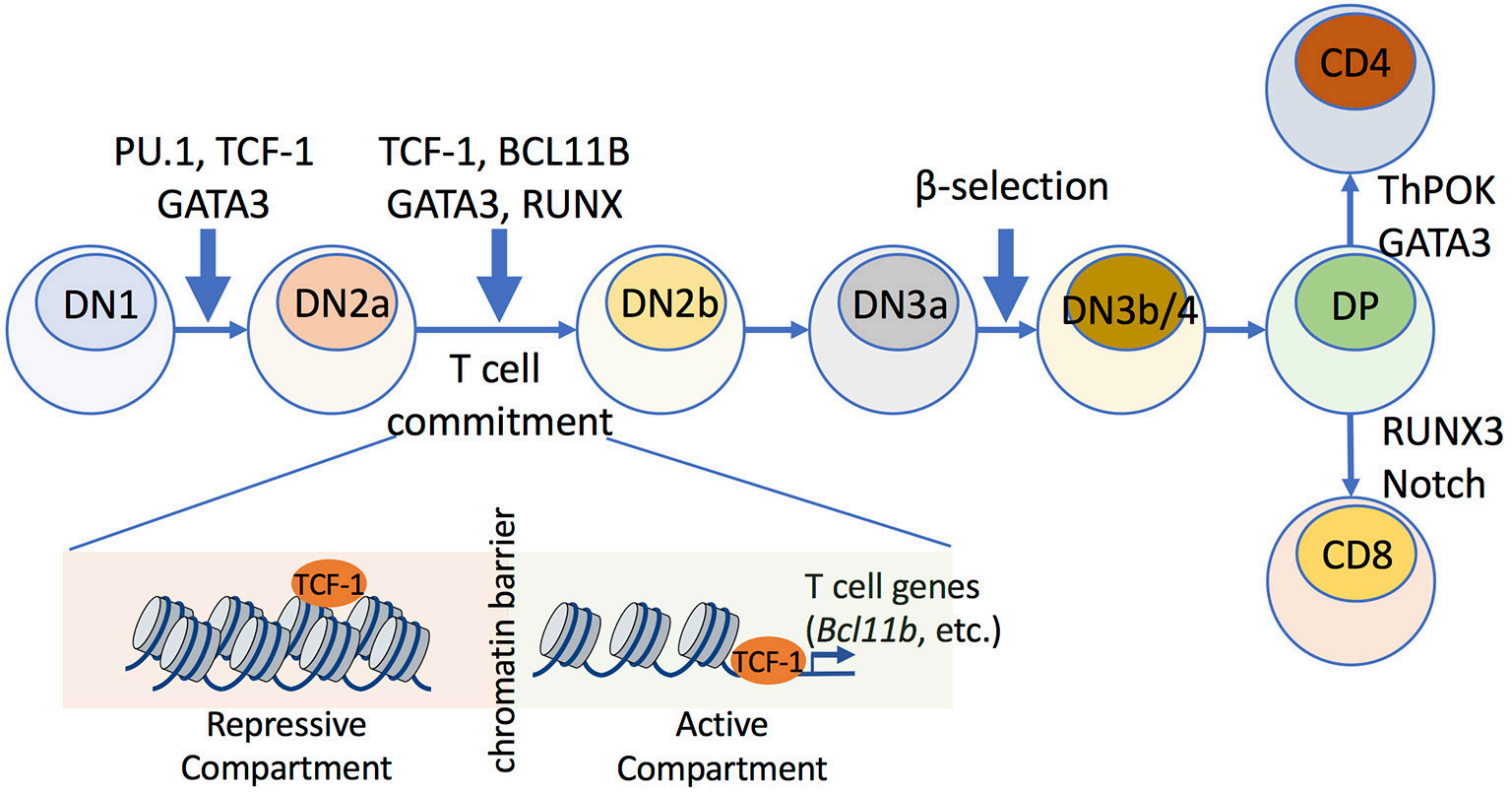
Transcription factors that mediate chromatin accessibility during early thymic T cell development. Multiple TFs play roles in early stages of T cell maturation, which involve commitment of hematopoietic stem cells to T cell progenitors. The early DN stage consists of DN1, DN2a/b, DN3a/b, and DN4 cells. During T cell commitment, which occurs between the DN2a and DN2b stages, TCF-1 establishes chromatin accessibility and mediates compartment switch, where repressive compartments that harbor T cell-lineage-specific genes (e.g., *Bcl11b*) are switched to transcriptionally active compartments. TCF-1 upregulates the expression of BCL11B, which further remodels chromatin architecture and stabilizes the intra-TAD contacts within mature T cell subsets.

Lineage specific transcription factors (LSTFs) or master regulators are expressed at critical times during lymphoid development or differentiation, and they contribute to cell type determination. During the development of T cells in the thymus, master regulators such as TCF-1, BCL11B, GATA3, PU.1, and RUNX family TFs are critical for T-lineage commitment (Figure 1) (1–3). To properly regulate gene expression, TFs must recognize and bind to their sequence-specific DNA binding sites (motifs). Access to regulatory regions in the genome is tightly controlled by chromatin structure. Genome-wide analysis using Hi-C technology, a method coupling 3C (Chromosome Conformation Capture) methodology with high-throughput DNA sequencing (4), has revealed that the genome can be divided into spatially separated regions or “compartments,” which are composed of smaller Topologically Associating Domains (TADs) that can be brought into close proximity to each other by chromatin looping (4–6).

During early T cell development, hematopoietic stem cells develop into T cell progenitor cells, termed CD4^−^CD8^−^ or double-negative (DN) thymocytes, which can then progress through four stages of maturation (denoted DN1, DN2a/b, DN3a/b, and DN4 cells). T cell commitment occurs at the DN2a to DN2b developmental transitional stages (1–3), and there is a key checkpoint termed β-selection at the CD25^+^CD44^−^ DN3a to DN3b/4 maturation step, with gene rearrangement of the TCR β chain. Following β-selection, T cells further mature into CD4^+^CD8^+^ double-positive (DP) cells, which express both CD4 and CD8 (3). Some evidence suggests that dynamic changes in chromatin modifications and transcription are associated with T cell development (7), but it is not clear if there are genome-wide modifications in higher-order chromatin structures and whether such structures are required to establish T cell identity. GATA-3 is essential throughout the early T cell developmental stages, including for T cell commitment, β-selection, and CD4^+^ cell fate choice during positive selection (8).

The IL-7/IL-7R axis plays major roles in the survival of DN thymocytes during early T-cell development (9, 10). IL-7-signaling activates major signaling pathways, including JAK1/JAK3-STAT5 and PI 3-kinase, and Y449 of the IL-7R is part of a YxxM motif and can mediate not only recruitment of STAT5 but also the p85 subunit of PI 3-kinase (11). IL-7-mediated signaling results in the induction of anti-apoptotic BCL-2 and MCL-1 proteins but the decreased expression of pro-apoptotic proteins (9), and STAT5 has been implicated in the regulation of expression of BCL-2 (12). IL-7-mediated STAT5 activation controls chromatin accessibility and rearrangement of the TCRγ locus (13, 14). In addition to its activation of STAT5, IL-7 was reported to activate NFATc1, with this serving as an alternative signaling pathway that cooperates with STAT5 to guide thymocyte development (15). Thus, IL-7-mediated transcriptional activation serves important roles in T cell development.

Recent studies have provided further insights into the mechanisms by which two TFs, TCF-1, and BCL11B, drive T cell differentiation by modifying the nuclear architecture to generate distinct chromatin landscapes (16, 17). Chromatin accessibility across distinct stages of T cell development was profiled using single-cell DNase-Seq (DNase I hypersensitive sites sequencing) (18, 19) and ATAC-Seq (Assay for Transposase Accessible Chromatin combined with DNA sequencing) (20, 21) to reveal that dynamic modifications in chromatin accessibility appeared genome-wide during T cell differentiation (16, 17). Strikingly, different stages of chromatin accessibility were observed as developing cells progress during T cell commitment (Figure 1). BCL11B, a critical regulator of T cell commitment, was found to play critical roles in maintaining higher-order chromatin structures and was associated with increased chromatin interactions during T cell lineage commitment (17). Furthermore, at early stages of T cell differentiation, TCF-1 was significantly enriched at accessible chromatin that was associated with T cell-lineage-specific gene loci (16). Mice deficient in *Tcf7*, which encodes TCF-1, cannot properly establish the open chromatin landscape of normal T cells, suggesting that the initiation of chromatin remodeling was TCF-1-dependent, and this was particularly evident at the *Bcl11b* locus (Figure 1).

PU.1 (encoded by *Spi1*/*Sfpi1*) was shown to function as a specialized nucleosome-binding transcription factor during the DN1-DN2 transition (22, 23), and this factor can bind to closed chromatin and rapidly open genomic sites. Specifically, ChIP-Seq (Chromatin Immunoprecipitation combined with high-throughput DNA sequencing) (24, 25) and ATAC-Seq analysis showed that the chromatin of selected regions is opened by PU.1 within 24 h (22, 23), suggesting that PU.1 acts as a “pioneer factor” to remodel chromatin structure during early T-cell development.

## Transcription factors that mediate chromatin accessibility during T cell differentiation

**Figure 2:**
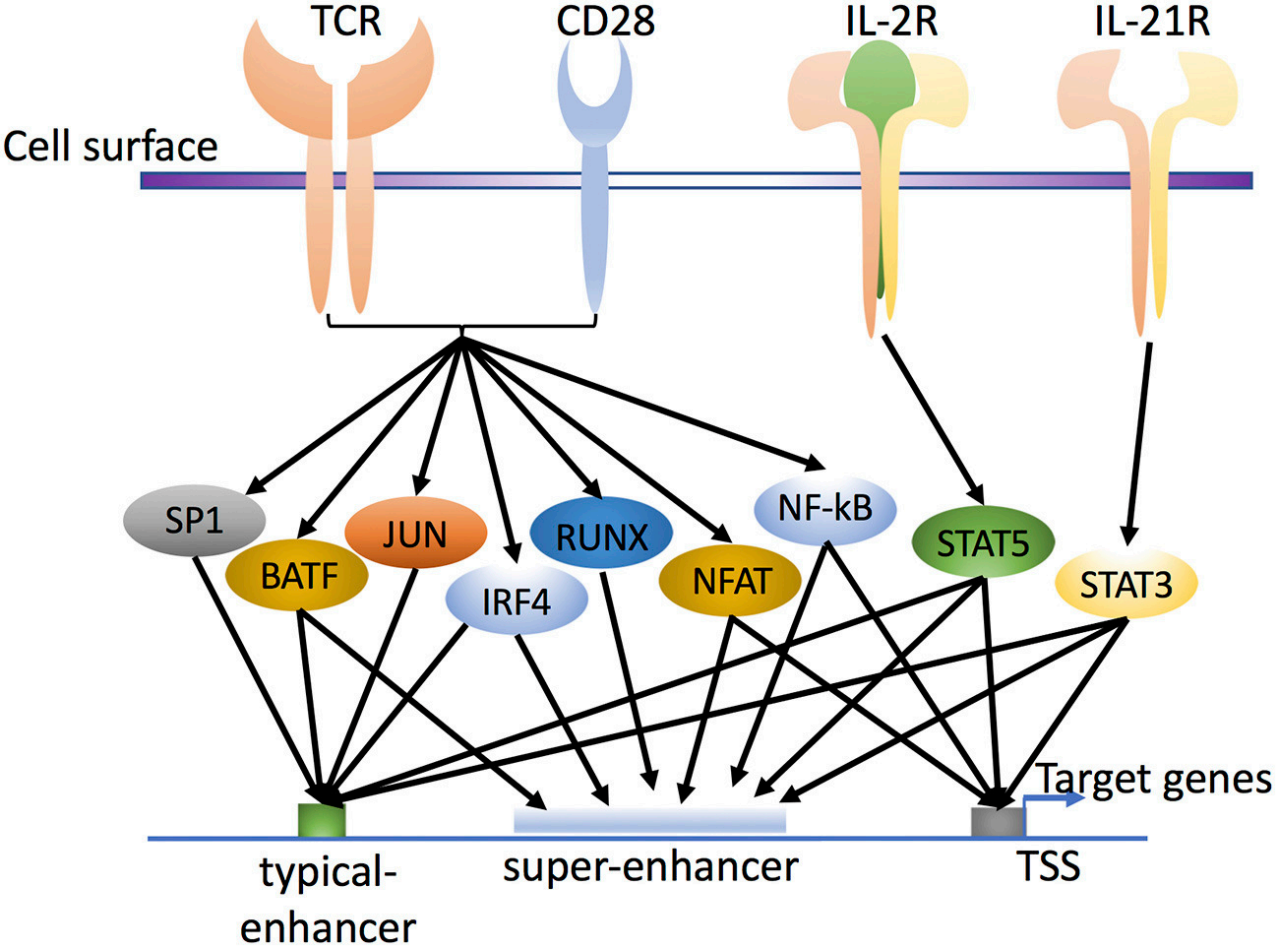
Transcription factors in T cell activation and differentiation. T cell receptor (TCR) and CD28 signaling activate various pioneer factors, such as NF-κB, NFAT, and AP-1 (FOS and JUN family proteins; the schematic shows BATF as the FOS-like AP-1 family protein). In addition, cytokine stimulation activates cytokine-specific TFs, such as IL-2-activated STAT5 and IL-21-activated STAT3, through their own cytokine receptors IL-2R and IL-21R, respectively. Together, these factors influence the enhancer landscape in a genome-wide fashion, with binding of TFs to typical-enhancers and/or super-enhancers to regulate the expression of target genes in T cells and influence cell differentiation and cell plasticity. Whereas typical enhancers span more limited regions, super-enhancers include groups, or clusters of enhancer elements, that span broader regions and are densely bound by transcriptional co-activators.

During CD4^+^ T cell differentiation, a range of pioneer factors are activated to shape the epigenetic landscape and regulate chromatin accessibility for TFs (26–29). T cell activation requires antigen signaling via the TCR and co-stimulation with CD28, resulting in nuclear translocation of a number of TFs, including AP-1 and NFAT (Figure 2). Interestingly, BATF, a FOS-like AP-1 family transcription factor, and IRF4 were shown to function as pioneer factors that could regulate chromatin accessibility during differentiation of Th17 (30) and CD8^+^ T cells (31).

Following TCR stimulation, cytokines including IL-2 and IL-21 are also produced and in turn activate and induce the nuclear translocation of STAT proteins (e.g., STAT5 and STAT3) through cytokine receptors IL-2R and IL-21R (Figure 2), and these factors collectively help to prepare the T cell chromatin landscape. Interestingly, both STAT dimers and tetramers can form, with STAT5 tetramers being critical for the normal development and expansion of key immune populations (32, 33). Cytokines secreted by immune cells can also drive T helper cell differentiation. For example, IL-12, IL-4, and IL-6 drive Th1, Th2, and Th17 differentiation, respectively, with critical roles for IL-2 in promoting (Th1, Th2, Th9, Treg) or inhibiting (Th17 or T follicular helper [Tfh] cell) differentiation and often opposing actions for IL-21 (34–37). All of these cytokines are 4-alpha helical bundle type 1 cytokines that use the JAK-STAT pathway as a major signaling pathway to transduce extracellular cytokine signals into the cell and regulate expression of corresponding genes (37–39). Interestingly, of these cytokines, IL-2, IL-4, and IL-21 signal via receptors that belong to the common cytokine receptor γ chain (γ_c_, also known as the IL-2 receptor γ chain IL-2Rγ, or CD132) family of cytokines (40, 41). During CD4^+^ T cell differentiation, STATs can have major impact on the activation of lineage-specific enhancers and the suppression of enhancers associated with alternative cell fates. For example, STATs can shape the active enhancer landscape in Th1 and Th2 cells (42, 43) in the presence of different cytokine signals, with IL-12/STAT4 and IFN-γ/STAT1 driving Th1 and IL-4/STAT6 driving Th2 differentiation, respectively. In addition, however, IL-2 via STAT5 serves a key role and primes T cells for responsiveness to IL-12 and IL-4 and Th differentiation. For Th1 differentiation, IL-2 via STAT5 augments expression of IL-12Rβ2 and TBET (44) and for Th2 differentiation, it augments expression of IL-4Rα (45) and IL-4 (46–48), with IL-2-induced STAT5 kinetically binding earlier to the *Il4ra* than to the *Il4* locus. Interestingly, IL-2-activated STAT5 binding was shown to augment chromatin accessibility at the *Il4* locus (46). IL-2 via STAT5 also inhibits Th17 differentiation (49, 50), potentially by several mechanisms, including a direct IL-2-STAT5 competition with IL-6-STAT3 (49, 51), the inhibition by IL-2-STAT5 of gp130 expression and by IL-2-mediated induction of TBET, which interacts with RUNX1, potentially limiting the required RUNX1-RORγt interaction (44). Moreover, IL-2-STAT5 drives Th9 differentiation (52) and limits Tfh differentiation (53, 54) whereas, IL-21-STAT3 has an opposing effect (52, 55, 56). As compared to pioneer factors, cytokines that influence Th differentiation have less profound effects on the epigenetic landscape.

## Interplay of chromatin and transcription factors AP-1, IRF4, and STAT3 in T cells

**Figure 3:**
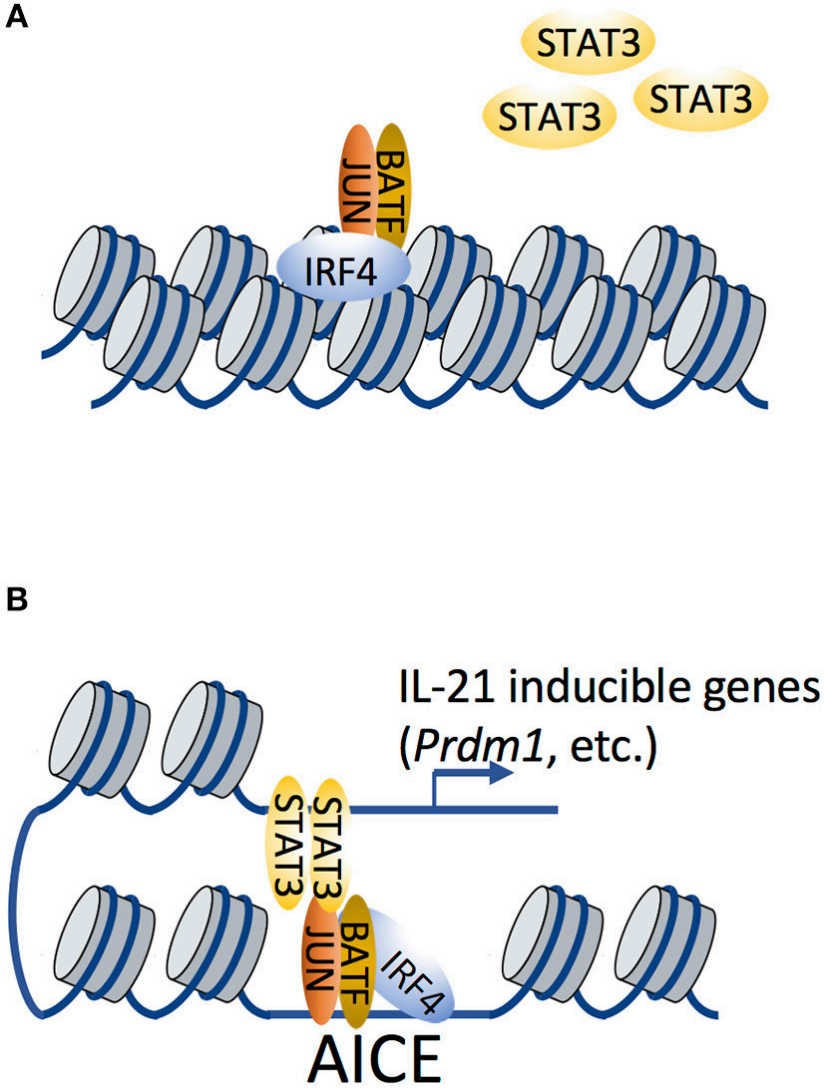
IRF4 and BATF remodel the chromatin landscape to facilitate subsequent recruitment of transcription factor STAT3**. (A)** Cooperative binding of AP-1 (shown here as a BATF-JUN heterodimer; BATF in this setting is the FOS-like factor) and IRF4 function as pioneer factors to remodel the chromatin landscape, therefore affecting chromatin accessibility. **(B)** STAT3 is subsequently recruited by AP-1/IRF4 complexes (which recognize AP-1-IRF composite elements, AICEs) via possible STAT3-JUN interactions. Such STAT3/AP-1/IRF4 complexes have been shown to regulate the expression of IL-21-inducible genes.

BATF and IRF4 were shown to functionally cooperate and recognize specific AP-1-IRF composite elements (AICEs) mainly in T cells and dendritic cells (57–59) and these factors cooperate to regulate chromatin accessibility during the differentiation of Th17 (30) and CD8^+^ T (31) cells (Figure 3). FAIRE-Seq (Chromatin accessibility analysis using formaldehyde-assisted isolation of regulatory elements sequencing) (60) revealed that the loss of BATF or IRF4 in Th0 or Th17 cells had little if any effect on genomic loci already accessible in naive cells, but most loci with inducible accessibility exhibited marked reductions in *Batf*- or *Irf4*-deficient mice compared to wild-type cells, suggesting that IRF4 and BATF remodel the chromatin landscape and potentially facilitate subsequent recruitment of TFs involved in regulating expression of Th17-relevant genes (30). Enhancer occupancy by AP-1/IRF4 complexes correlates with sensitivity of gene expression in response to TCR signaling (61, 62), so that genes with low-affinity or high-affinity AICE-dependent enhancers are induced at lower or higher TCR signal strength, respectively. IRF4 alone was also shown to be induced in a manner dependent on TCR affinity, and as a dose-dependent regulator of the metabolic function of activated T cells (63). BATF is also a key regulator of early effector CD8^+^ T cell differentiation (31), and BATF-deficient CD8^+^ T cells are profoundly defective in their ability to undergo naive to effector differentiation and proliferative expansion. Moreover, BATF and IRF1 are induced early during *in vitro* regulatory T (Treg) cell differentiation and act as pioneer factors for the differentiation of type 1 Treg (Tr1) cells (64). BACH2, like AP-1 factors that contain a bZIP domain, can regulate CD8^+^ T cell differentiation by controlling the access of AP-1 factors to enhancers, thus limiting the expression of TCR-driven genes by attenuating the availability of AP-1 sites to JUN family TFs (65, 66).

IRF4 often cooperates with STAT3 in modulating IL-21-dependent gene expression in Tfh and Th17 cells (30, 67, 58). Given that STAT3 can physically bind to c-JUN (68), it is reasonable to hypothesize that STAT3 can be recruited by BATF-JUN-IRF4 complexes via STAT3-JUN interactions (Figure 3). ChIP-Seq analysis revealed that IL-21-induced STAT3 binding was dramatically diminished in *Irf4*^−/−^ CD4^+^ T cells compared to WT cells (67), suggesting that it was IRF4-dependent. It is possible that STAT3 directly binds to IRF4, or that its binding is dependent on chromatin accessibility that is pre-patterned by IRF4 to facilitate the subsequent recruitment of STAT3 to AP-1. However, STAT3 binding motifs are not enriched in genomic proximity to AICEs (58, 69), suggesting that STAT3-IRF4 association may also occur via long-range chromatin interactions, a hypothesis that remains to be experimentally validated.

## T cell responses to different stimuli including IL-2 and IL-21 and the role of super-enhancers

After antigen encounter, CD4^+^ T cells are activated and secrete cytokines including IL-2 and IL-21, which regulate immune cell differentiation and effector functions by differentially activating specific STAT proteins that recognize and bind to γ-interferon-activated sequence (GAS) motifs. IL-2 potently activates STAT5, whereas IL-21 primarily activates STAT3. This differential STAT activation leads to differential gene expression by these cytokines. It is established that STAT proteins are critical components of cytokine-activated enhancers, but recently their roles related to super-enhancers (70) and their abilities to fine-tune gene expression (71) have been elucidated, with, for example, greater IL-2-inducibility of genes with STAT5-based super-enhancers, as compared to STAT5-based typical enhancers (71). As opposed to typical enhancers, where factor binding occurs in more limited regions, super-enhancers (also known as stretched or clustered enhancers) (70, 72, 73) represent groups of putative enhancers in close genomic proximity that span broader regions (Figure 2), are densely bound by transcriptional coactivators, and usually are associated with high levels of the active chromatin mark histone H3 lysine 27 acetylation (H3K27Ac). Although super-enhancers were originally recognized in the setting of master regulator genes (74) and genes associated with cell identity, STAT5- and STAT3-dependent super-enhancers have now also been shown to exist and to regulate gene expression in a cytokine- and context-specific manner (71). Chromatin interaction analysis using paired-end tag sequencing (ChIA-PET) (75, 76) revealed that IL-2–activated STAT5 can influence RNA Polymerase II (RNA Pol II)-based chromatin interactions, with looping anchor sites in proximity to STAT5 binding sites. Moreover, CRISPR–Cas9 (77, 78) genome editing was used to generate mutant mice in order to functionally analyze the STAT5-bound super-enhancer containing gene, *Il2ra, in vivo*. When three of the super-enhancer elements were separately deleted, each exhibited defective expression of IL-2Rα, indicating that each enhancer element contributed to IL-2-induced IL-2Rα expression and that these elements were not functionally redundant (71). These observations provide insights into the mechanism underlying the regulation of IL-2 target genes. Interestingly, IL-2-based super-enhancers included not only positive regulators of signaling, such as *Il2ra*, but also negative regulators such as SOCS family proteins (e.g., *Cish*), revealing that super-enhancers are critical for both the positive and negative regulation of IL-2 signaling (71).

Similar to the mouse *Il2ra* gene, human *IL2RA* also has a similar super-enhancer that is densely bound by STAT5, and some of the enhancer elements are highly conserved in both mouse and human, consistent with an evolutionarily conserved mode of gene regulation (71, 79, 80). Interestingly, tiled CRISPR activation (CRISPRa) (81) was used to identify several CRISPRa-responsive elements with chromatin features of stimulus-responsive enhancers, including an *IL2RA* enhancer that contains a non-coding autoimmunity risk variant (80) that is conserved between humans and mice. Mutating this element in mice did not completely block *Il2ra* gene expression but rather delayed gene activation in response to TCR stimulation, indicating that the kinetics of *Il2ra* gene expression are important. This mutation skewed polarization of naive T cells from Treg cells toward pro-inflammatory Th17 cells, which elucidates its role in autoimmune disease (80).

## Enhancer-promoter interactions and autoimmune disease-associated SNPs

**Figure 4:**
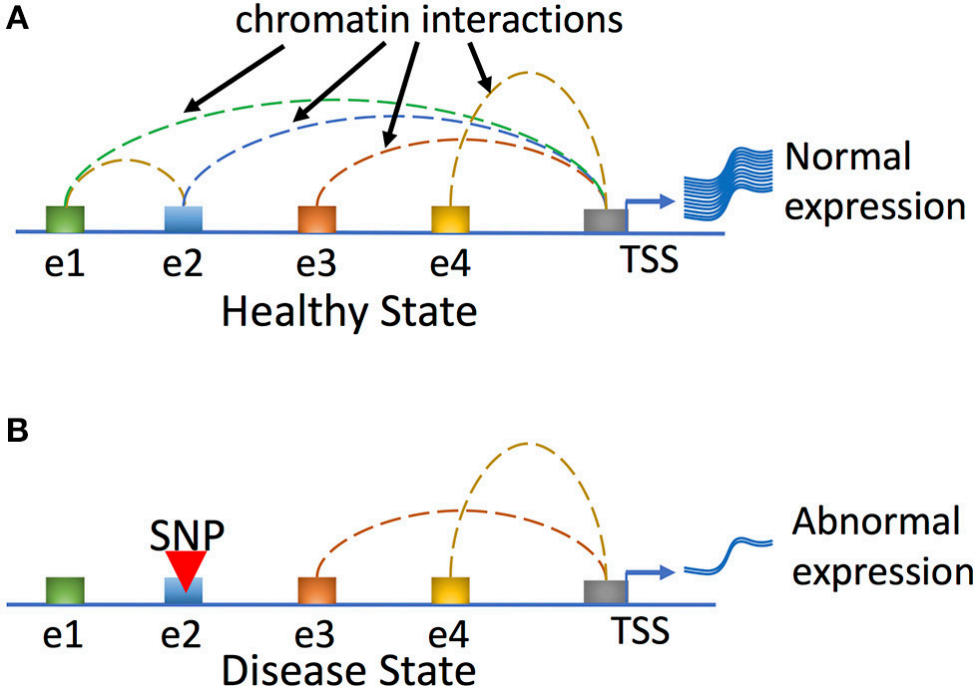
Genetic variation can affect human disease phenotypes by disrupting regulatory networks mediated by long-range chromatin interactions. In the healthy state **(A)**, all regulatory enhancer elements (shown here for a hypothetical gene as elements e1–e4) are utilized and loop to the promoter to effect normal gene expression. In the disease state **(B)**, a hypothetical genetic variant (SNP) residing at e2 disrupts enhancer-promoter and enhancer-enhancer interactions and results in abnormal gene expression and disease phenotypes.

Gene expression is regulated via complex interactions between promoters and long-range regulatory elements, and disruption of chromatin interactions by mutations (e.g., SNPs or INDELs) may result in altered target gene expression that leads to disease development (Figure 4). Another study correlated histone modification of H3K27ac with active enhancers and promoters and furthermore analyzed protein-centric chromatin interactions by utilizing HiChIP, chromatin immunoprecipitation (ChIP) and Hi-C assays (82). By generating enhancer–promoter contacts in primary naive CD4^+^ T cells, Treg cells, and Th17 cells, chromatin loops were identified that were shared by all three cell types (82). Strikingly, the majority of these chromatin interaction anchors were associated with enhancers or promoters. Furthermore, autoimmune disease–associated variants in intergenic regions could interact with multiple target genes, providing insights into the functional interrogation of disease associated genetic variants; however, further high-resolution chromatin interactions in various cell types are needed to better explain how connections between variants and genes can be translated into molecular and cellular functions.

## Concluding comments

In summary, studies of the transcriptional and epigenetic regulation of T cells have identified several mechanisms of cross-regulation between TFs, chromatin modifiers, and the pre-existing chromatin landscape. The interactions between chromatin and TFs are influenced by a range of stimuli, including TCR and cytokine signals. Transcription factors are important for cell function, and they collaborate combinatorically with other factors to influence gene regulation. Their binding to DNA depends on epigenetic landscapes, and their function may depend on chromatin interactions to juxtapose distal regulatory elements with gene promoters. The ability of cytokine-activated proteins to modify nucleosome packing and influence histone modifications allows them to control developmental processes. The gene regulatory networks that determine T cell development are broad and involve chromatin accessibility, epigenetic status, and distant chromatin interactions in both time- and context-dependent manners. Our evolving understanding of gene regulatory networks will help to comprehensively link genetic variants to putative gene targets, furthering our understanding of molecular mechanisms for a range of immune diseases. Achieving a deeper understanding of the mechanisms involved has now been greatly facilitated by genetic manipulations including CRISPR/Cas9 gene editing but still awaits other advances, such as the ability to comprehensively study single cells in real time.

## Author contributions

All authors listed have made a substantial, direct and intellectual contribution to the work, and approved it for publication.

### Conflict of interest statement

The authors declare that the research was conducted in the absence of any commercial or financial relationships that could be construed as a potential conflict of interest. The handling Editor declared a shared affiliation, though no other collaboration, with the authors.

## References

[1] Rothenberg EV. The chromatin landscape and transcription factors in T cell programming. Trends Immunol. (2014) 35:195–204. 10.1016/j.it.2014.03.001 24703587 PMC4039984

[2] Yui MA Rothenberg EV. Developmental gene networks: a triathlon on the course to T cell identity. Nat Rev Immunol. (2014) 14:529–45. 10.1038/nri3702 25060579 PMC4153685

[3] Hosokawa H Rothenberg EV. Cytokines, transcription factors, and the initiation of T-Cell development. Cold Spring Harb Perspect Biol. (2018) 10:a028621. 10.1101/cshperspect.a028621 28716889 PMC5876153

[4] Lieberman-Aiden E van Berkum NL Williams L Imakaev M Ragoczy T Telling A. Comprehensive mapping of long-range interactions reveals folding principles of the human genome. Science (2009) 326:289–93. 10.1126/science.1181369 19815776 PMC2858594

[5] Rao SS Huntley MH Durand NC Stamenova EK Bochkov ID Robinson JT. A 3D map of the human genome at kilobase resolution reveals principles of chromatin looping. Cell (2014) 159:1665–80. 10.1016/j.cell.2014.11.021 25497547 PMC5635824

[6] Dekker J Heard E. Structural and functional diversity of topologically associating domains. FEBS Lett. (2015) 589:2877–84. 10.1016/j.febslet.2015.08.044 26348399 PMC4598308

[7] Zhang JA Mortazavi A Williams BA Wold BJ Rothenberg EV. Dynamic transformations of genome-wide epigenetic marking and transcriptional control establish T cell identity. Cell (2012) 149:467–82. 10.1016/j.cell.2012.01.056 22500808 PMC3336965

[8] Hosoya T Maillard I Engel JD. From the cradle to the grave: activities of GATA-3 throughout T-cell development and differentiation. Immunol Rev. (2010) 238:110–25. 10.1111/j.1600-065X.2010.00954.x 20969588 PMC2965564

[9] Jiang Q Li WQ Aiello FB Mazzucchelli R Asefa B Khaled AR. Cell biology of IL-7, a key lymphotrophin. Cytok Growth Factor Rev. (2005) 16:513–33. 10.1016/j.cytogfr.2005.05.004 15996891

[10] Niu N Qin X. New insights into IL-7 signaling pathways during early and late T cell development. Cell Mol Immunol. (2013) 10:187–9. 10.1038/cmi.2013.11 23584490 PMC4012780

[11] Venkitaraman AR Cowling RJ. Interleukin-7 induces the association of phosphatidylinositol 3-kinase with the alpha chain of the interleukin-7 receptor. Eur J Immunol. (1994) 24:2168–74. 10.1002/eji.1830240935 7522165

[12] Li G Miskimen KL Wang Z Xie XY Brenzovich J Ryan JJ. STAT5 requires the N-domain for suppression of miR15/16, induction of bcl-2, and survival signaling in myeloproliferative disease. Blood (2010) 115:1416–24. 10.1182/blood-2009-07-234963 20008792 PMC2826763

[13] Ye SK Agata Y Lee HC Kurooka H Kitamura T Shimizu A. The IL-7 receptor controls the accessibility of the TCRgamma locus by Stat5 and histone acetylation. Immunity (2001) 15:813–23. 10.1016/S1074-7613(01)00230-8 11728342

[14] Wagatsuma KS Tani-ichi S Liang B Shitara S Ishihara K Abe M. STAT5 orchestrates local epigenetic changes for chromatin accessibility and rearrangements by direct binding to the TCRgamma locus. J Immunol. (2015) 195:1804–14. 10.4049/jimmunol.1302456 26195811

[15] Patra AK Avots A Zahedi RP Schuler T Sickmann A Bommhardt U. An alternative NFAT-activation pathway mediated by IL-7 is critical for early thymocyte development. Nat Immunol. (2013) 14:127–35. 10.1038/ni.2507 23263556

[16] Johnson JL Georgakilas G Petrovic J Kurachi M Cai S Harly C. Lineage-determining transcription factor TCF-1 initiates the epigenetic identity of t cells. Immunity (2018) 48:243–57 e10. 10.1016/j.immuni.2018.01.012 29466756 PMC5824646

[17] Hu G Cui K Fang D Hirose S Wang X Wangsa D. Transformation of accessible chromatin and 3D nucleome underlies lineage commitment of early T cells. Immunity (2018) 48:227–42 e8. 10.1016/j.immuni.2018.01.013 29466755 PMC5847274

[18] Boyle AP Davis S Shulha HP Meltzer P Margulies EH Weng Z. High-resolution mapping and characterization of open chromatin across the genome. Cell (2008) 132:311–22. 10.1016/j.cell.2007.12.014 18243105 PMC2669738

[19] Song L Crawford GE. DNase-seq: a high-resolution technique for mapping active gene regulatory elements across the genome from mammalian cells. Cold Spring Harb Protoc. (2010) 2010:pdbprot5384. 10.1101/pdb.prot5384 20150147 PMC3627383

[20] Buenrostro JD Giresi PG Zaba LC Chang HY Greenleaf WJ. Transposition of native chromatin for fast and sensitive epigenomic profiling of open chromatin, DNA-binding proteins and nucleosome position. Nat Methods (2013) 10:1213–8. 10.1038/nmeth.2688 24097267 PMC3959825

[21] Buenrostro JD Wu B Chang HY Greenleaf WJ. ATAC-seq: a method for assaying chromatin accessibility genome-wide. Curr Protoc Mol Biol. (2015) 109:21.29.1–9. 10.1002/0471142727.mb2129s109 25559105 PMC4374986

[22] Hosokawa H Ungerback J Wang X Matsumoto M Nakayama KI Cohen SM. Transcription factor PU.1 represses and activates gene expression in early T cells by redirecting partner transcription factor binding. Immunity (2018) 48:1119–34 e7. 10.1016/j.immuni.2018.04.024 29924977 PMC6063530

[23] Ungerback J Hosokawa H Wang X Strid T Williams BA Sigvardsson M. Pioneering, chromatin remodeling, and epigenetic constraint in early T-cell gene regulation by SPI1 (PU.1). Genome Res. (2018) 28:1508–19. 10.1101/gr.231423.117 30171019 PMC6169891

[24] Barski A Cuddapah S Cui K Roh TY Schones DE Wang Z. High-resolution profiling of histone methylations in the human genome. Cell (2007) 129:823–37. 10.1016/j.cell.2007.05.009 17512414

[25] Johnson DS Mortazavi A Myers RM Wold B. Genome-wide mapping of *in vivo* protein-DNA interactions. Science (2007) 316:1497–502. 10.1126/science.1141319 17540862

[26] Josefowicz SZ. Regulators of chromatin state and transcription in CD4 T-cell polarization. Immunology (2013) 139:299–308. 10.1111/imm.12115 23590627 PMC3701176

[27] Tripathi SK Lahesmaa R. Transcriptional and epigenetic regulation of T-helper lineage specification. Immunol Rev. (2014) 261:62–83. 10.1111/imr.12204 25123277 PMC4255756

[28] Soufi A Donahue G Zaret KS. Facilitators and impediments of the pluripotency reprogramming factors' initial engagement with the genome. Cell (2012) 151:994–1004. 10.1016/j.cell.2012.09.045 23159369 PMC3508134

[29] Zaret KS Carroll JS. Pioneer transcription factors: establishing competence for gene expression. Genes Dev. (2011) 25:2227–41. 10.1101/gad.176826.111 22056668 PMC3219227

[30] Ciofani M Madar A Galan C Sellars M Mace K Pauli F. A validated regulatory network for Th17 cell specification. Cell (2012) 151:289–303. 10.1016/j.cell.2012.09.016 23021777 PMC3503487

[31] Kurachi M Barnitz RA Yosef N Odorizzi PM DiIorio MA Lemieux ME. The transcription factor BATF operates as an essential differentiation checkpoint in early effector CD8+ T cells. Nat Immunol. (2014) 15:373–83. 10.1038/ni.2834 24584090 PMC4000237

[32] Lin JX Du N Li P Kazemian M Gebregiorgis T Spolski R. Critical functions for STAT5 tetramers in the maturation and survival of natural killer cells. Nat Commun. (2017) 8:1320. 10.1038/s41467-017-01477-5 29105654 PMC5673064

[33] Lin JX Li P Liu D Jin HT He J Ata Ur Rasheed M. Critical Role of STAT5 transcription factor tetramerization for cytokine responses and normal immune function. Immunity (2012) 36:586–99. 10.1016/j.immuni.2012.02.017 22520852 PMC3551341

[34] Zhu J Paul WE. Peripheral CD4+ T-cell differentiation regulated by networks of cytokines and transcription factors. Immunol Rev. (2010) 238:247–62. 10.1111/j.1600-065X.2010.00951.x 20969597 PMC2975272

[35] Zhu J Yamane H Paul WE. Differentiation of effector CD4 T cell populations (^*^). Annu Rev Immunol. (2010) 28:445–89. 10.1146/annurev-immunol-030409-101212 20192806 PMC3502616

[36] Liao W Lin JX Leonard WJ. IL-2 family cytokines: new insights into the complex roles of IL-2 as a broad regulator of T helper cell differentiation. Curr Opin Immunol. (2011) 23:598–604. 10.1016/j.coi.2011.08.003 21889323 PMC3405730

[37] Lin JX Leonard WJ. The common cytokine receptor gamma chain family of cytokines. Cold Spring Harb Perspect Biol. (2017) 10:a028449. 10.1101/cshperspect.a028449 29038115 PMC6120701

[38] Leonard WJ O'Shea JJ. Jaks and STATs: biological implications. Annu Rev Immunol. (1998) 16:293–322. 10.1146/annurev.immunol.16.1.293 9597132

[39] O'Shea JJ Plenge R. JAK and STAT signaling molecules in immunoregulation and immune-mediated disease. Immunity (2012) 36:542–50. 10.1016/j.immuni.2012.03.014 22520847 PMC3499974

[40] Noguchi M Nakamura Y Russell SM Ziegler SF Tsang M Cao X. Interleukin-2 receptor gamma chain: a functional component of the interleukin-7 receptor. Science (1993) 262:1877–80. 10.1126/science.8266077 8266077

[41] Russell SM Keegan AD Harada N Nakamura Y Noguchi M Leland P. Interleukin-2 receptor gamma chain: a functional component of the interleukin-4 receptor. Science (1993) 262:1880–3. 10.1126/science.8266078 8266078

[42] Vahedi GC Poholek A Hand TW Laurence A Kanno Y O'Shea JJ. Helper T-cell identity and evolution of differential transcriptomes and epigenomes. Immunol Rev. (2013) 252:24–40. 10.1111/imr.12037 23405893 PMC3577092

[43] Vahedi G Takahashi H Nakayamada S Sun HW Sartorelli V Kanno Y. STATs shape the active enhancer landscape of T cell populations. Cell (2012) 151:981–93. 10.1016/j.cell.2012.09.044 23178119 PMC3509201

[44] Liao W Lin JX Wang L Li P Leonard WJ. Modulation of cytokine receptors by IL-2 broadly regulates differentiation into helper T cell lineages. Nat Immunol. (2011) 12:551–9. 10.1038/ni.2030 21516110 PMC3304099

[45] Liao W Schones DE Oh J Cui Y Cui K Roh TY. Priming for T helper type 2 differentiation by interleukin 2-mediated induction of interleukin 4 receptor alpha-chain expression. Nat Immunol. (2008) 9:1288–96. 10.1038/ni.1656 18820682 PMC2762127

[46] Cote-Sierra J Foucras G Guo L Chiodetti L Young HA Hu-Li J. Interleukin 2 plays a central role in Th2 differentiation. Proc Natl Acad Sci USA. (2004) 101:3880–5. 10.1073/pnas.0400339101 15004274 PMC374338

[47] Zhu J Cote-Sierra J Guo L Paul WE. Stat5 activation plays a critical role in Th2 differentiation. Immunity (2003) 19:739–48. 10.1016/S1074-7613(03)00292-9 14614860

[48] Zhu J Yamane H Cote-Sierra J Guo L Paul WE. GATA-3 promotes Th2 responses through three different mechanisms: induction of Th2 cytokine production, selective growth of Th2 cells and inhibition of Th1 cell-specific factors. Cell Res. (2006) 16:3–10. 10.1038/sj.cr.7310002 16467870

[49] Laurence A Tato CM Davidson TS Kanno Y Chen Z Yao Z. Interleukin-2 signaling via STAT5 constrains T helper 17 cell generation. Immunity (2007) 26:371–81. 10.1016/j.immuni.2007.02.009 17363300

[50] Stockinger B Good for goose, but not for gander: IL-2 interferes with Th17 differentiation.Immunity (2007) 26:278–9. 10.1016/j.immuni.2007.03.001 17376391

[51] Yang XP Ghoreschi K Steward-Tharp SM Rodriguez-Canales J Zhu J Grainger JR. Opposing regulation of the locus encoding IL-17 through direct, reciprocal actions of STAT3 and STAT5. Nat Immunol. (2011) 12:247–54. 10.1038/ni.1995 21278738 PMC3182404

[52] Liao W Spolski R Li P Du N West EE Ren M. Opposing actions of IL-2 and IL-21 on Th9 differentiation correlate with their differential regulation of BCL6 expression. Proc Natl Acad Sci USA. (2014) 111:3508–13. 10.1073/pnas.1301138111 24550509 PMC3948278

[53] Johnston RJ Choi YS Diamond JA Yang JA Crotty S. STAT5 is a potent negative regulator of TFH cell differentiation. J Exp Med. (2012) 209:243–50. 10.1084/jem.20111174 22271576 PMC3281266

[54] Nurieva RI Podd A Chen Y Alekseev AM Yu M Qi X. STAT5 protein negatively regulates T follicular helper (Tfh) cell generation and function. J Biol Chem. (2012) 287:11234–9. 10.1074/jbc.M111.324046 22318729 PMC3322890

[55] Ma CS Avery DT Chan A Batten M Bustamante J Boisson-Dupuis S. Functional STAT3 deficiency compromises the generation of human T follicular helper cells. Blood (2012) 119:3997–4008. 10.1182/blood-2011-11-392985 22403255 PMC3355712

[56] Nurieva RI Chung Y Hwang D Yang XO Kang HS Ma L Generation of T follicular helper cells is mediated by interleukin-21 but independent of T helper **1**:2, or 17 cell lineages. Immunity (2008) 29:138–49. 10.1016/j.immuni.2008.05.009 18599325 PMC2556461

[57] Glasmacher E Agrawal S Chang AB Murphy TL Zeng W Vander Lugt B. A genomic regulatory element that directs assembly and function of immune-specific AP-1-IRF complexes. Science (2012) 338:975–80. 10.1126/science.1228309 22983707 PMC5789805

[58] Li P Spolski R Liao W Wang L Murphy TL Murphy KM. BATF-JUN is critical for IRF4-mediated transcription in T cells. Nature (2012) 490:543–6. 10.1038/nature11530 22992523 PMC3537508

[59] Tussiwand R Lee WL Murphy TL Mashayekhi M Kc W Albring JC. Compensatory dendritic cell development mediated by BATF-IRF interactions. Nature (2012) 490:502–7. 10.1038/nature11531 22992524 PMC3482832

[60] Giresi PG Kim J McDaniell RM Iyer VR Lieb JD. FAIRE (Formaldehyde-Assisted Isolation of Regulatory Elements) isolates active regulatory elements from human chromatin. Genome Res. (2007) 17:877–85. 10.1101/gr.5533506 17179217 PMC1891346

[61] Gallagher MP Berg LJ. Gene-enhancer variants reveal diverse TCR-mediated differentiation. Nat Immunol. (2017) 18:483–4. 10.1038/ni.3729 28418392

[62] Iwata A Durai V Tussiwand R Briseno CG Wu X Grajales-Reyes GE. Quality of TCR signaling determined by differential affinities of enhancers for the composite BATF-IRF4 transcription factor complex. Nat Immunol. (2017) 18:563–72. 10.1038/ni.3714 28346410 PMC5401770

[63] Man K Miasari M Shi W Xin A Henstridge DC Preston S. The transcription factor IRF4 is essential for TCR affinity-mediated metabolic programming and clonal expansion of T cells. Nat Immunol. (2013) 14:1155–65. 10.1038/ni.2710 24056747

[64] Karwacz K Miraldi ER Pokrovskii M Madi A Yosef N Wortman I. Critical role of IRF1 and BATF in forming chromatin landscape during type 1 regulatory cell differentiation. Nat Immunol. (2017) 18:412–21. 10.1038/ni.3683 28166218 PMC5901650

[65] Roychoudhuri R Clever D Li P Wakabayashi Y Quinn KM Klebanoff CA. BACH2 regulates CD8(+) T cell differentiation by controlling access of AP-1 factors to enhancers. Nat Immunol. (2016) 17:851–60. 10.1038/ni.3441 27158840 PMC4918801

[66] Sidwell T Kallies A. Bach2 is required for B cell and T cell memory differentiation. Nat Immunol. (2016) 17:744–5. 10.1038/ni.3493 27327995

[67] Kwon H Thierry-Mieg D Thierry-Mieg J Kim HP Oh J Tunyaplin C. Analysis of interleukin-21-induced Prdm1 gene regulation reveals functional cooperation of STAT3 and IRF4 transcription factors. Immunity (2009) 31:941–52. 10.1016/j.immuni.2009.10.008 20064451 PMC3272079

[68] Zhang X Wrzeszczynska MH Horvath CM Darnell JE Jr. Interacting regions in Stat3 and c-Jun that participate in cooperative transcriptional activation. Mol Cell Biol. (1999) 19:7138–46. 10.1128/MCB.19.10.7138 10490649 PMC84707

[69] Martinez GJ Rao A. Immunology. Cooperative transcription factor complexes in control. Science (2012) 338:891–2. 10.1126/science.1231310 23161983 PMC3621126

[70] Hnisz D Abraham BJ Lee TI Lau A Saint-Andre V Sigova AA. Super-enhancers in the control of cell identity and disease. Cell (2013) 155:934–47. 10.1016/j.cell.2013.09.053 24119843 PMC3841062

[71] Li P Mitra S Spolski R Oh J Liao W Tang Z Mo F. STAT5-mediated chromatin interactions in superenhancers activate IL-2 highly inducible genes: functional dissection of the Il2ra gene locus. Proc Natl Acad Sci USA. (2017) 114:12111–9. 10.1073/pnas.1714019114 29078395 PMC5699083

[72] Parker SC Stitzel ML Taylor DL Orozco JM Erdos MR Akiyama JA. Chromatin stretch enhancer states drive cell-specific gene regulation and harbor human disease risk variants. Proc Natl Acad Sci USA. (2013) 110:17921–6. 10.1073/pnas.1317023110 24127591 PMC3816444

[73] Pott S Lieb JD. What are super-enhancers? Nat Genet. (2015) 47:8–12. 10.1038/ng.3167 25547603

[74] Whyte WA Orlando DA Hnisz D Abraham BJ Lin CY Kagey MH. Master transcription factors and mediator establish super-enhancers at key cell identity genes. Cell (2013) 153:307–19. 10.1016/j.cell.2013.03.035 23582322 PMC3653129

[75] Li G Ruan X Auerbach RK Sandhu KS Zheng M Wang P. Extensive promoter-centered chromatin interactions provide a topological basis for transcription regulation. Cell (2012) 148:84–98. 10.1016/j.cell.2011.12.014 22265404 PMC3339270

[76] Tang Z Luo OJ Li X Zheng M Zhu JJ Szalaj P. CTCF-mediated human 3D genome architecture reveals chromatin topology for transcription. Cell (2015) 163:1611–27. 10.1016/j.cell.2015.11.024 26686651 PMC4734140

[77] Cong L Ran FA Cox D Lin S Barretto R Habib N. Multiplex genome engineering using CRISPR/Cas systems. Science (2013) 339:819–23. 10.1126/science.1231143 23287718 PMC3795411

[78] Mali P Yang L Esvelt KM Aach J Guell M DiCarlo JE. RNA-guided human genome engineering via Cas9. Science (2013) 339:823–6. 10.1126/science.1232033 23287722 PMC3712628

[79] Spolski R Li P Leonard WJ Biology and regulation of IL-2: from molecular mechanisms to human therapy. Nat Rev Immunol. (2018) 18:648–59. 10.1038/s41577-018-0046-y 30089912

[80] Simeonov DR Gowen BG Boontanrart M Roth TL Gagnon JD Mumbach MR. Discovery of stimulation-responsive immune enhancers with CRISPR activation. Nature (2017) 549:111–5. 10.1038/nature23875 28854172 PMC5675716

[81] Gilbert LA Horlbeck MA Adamson B Villalta JE Chen Y Whitehead EH. Genome-scale CRISPR-mediated control of gene repression and activation. Cell (2014) 159:647–61. 10.1016/j.cell.2014.09.029 25307932 PMC4253859

[82] Mumbach MR Satpathy AT Boyle EA Dai C Gowen BG Cho SW. Enhancer connectome in primary human cells identifies target genes of disease-associated DNA elements. Nat Genet. (2017) 49:1602–12. 10.1038/ng.3963 28945252 PMC5805393

